# The repaired man or the man with extras: medical human-cyborgs

**DOI:** 10.3389/fdgth.2026.1754061

**Published:** 2026-03-05

**Authors:** Gábor Speer

**Affiliations:** 1 Private Practitioner, Budapest, Hungary; 2 Semmelweis University Doctoral College, Budapest, Hungary

**Keywords:** artificial intelligence, cyborg, definition, human, posthuman

## Introduction

In this article, I aim to provide a definition of the term medical human-cyborg and explore its existence from humanistic and posthumanistic approaches to healthcare. I also aim to derive and define the term exclusively on the basis of real, existing medical technical solutions. Moreover, in order to reflect on the direction in which healthcare is heading and what the dangers of technological development might be. How did medicine move from implantation to medical human-cyborgs? Has it truly reached that stage?

The use of the term cyborg existence is linked to posthumanist philosophy. Donna J. Haraway's Cyborg Manifesto is one of the first posthumanist philosophical works (1). For Haraway, the cyborg is not about modifying the physical body; rather it is a metaphor for a mode of being completely permeated by technology—a society organized by technology. Her central question is the analysis of personality and identity integrated into technical networks. Haraway argues that in contemporary society, it is impossible to maintain distinctions of modern origin, such as human/non-human or organic/inorganic.

## Posthumanism in healthcare

Humanism proclaims the excellence of the human race, positioning it is as a unique summit in nature. However, posthumanism is present in almost all disciplines, including healthcare. Posthumanist expectations and proposals for change have been formulated primarily in nursing (2–4). Thus, the cyborg existence is not only an identity and a metaphor but also a possible attitude and a mode of being in healthcare.

Posthumanism encompasses several trends, unified by their rejection of inherent, purely human excellence (essence) (5). This does not imply the immediate acceptance of machines, but it does signify that “there is no clear dividing line between human and non-human” (6). It is a worldview without a center; however, this does not mean that it breaks with the human, but all its variants deny the existence of an autonomous human subject. Some posthumanists argue that the embeddedness of humanity in living and self-organizing matter positions the human as neither exclusively organizing nor exclusively organized (7).

There is no doubt that modern healthcare was conceived with humanistic goals. The right to health is clearly a humanistic idea. However, according to posthumanists, modern healthcare is only ostensibly the most humanistic of social institutions; its greatest falsehood is that it is equally accessible to all. In their article on posthumanist patient care, A.C. Laurin and P. Martin highlighted the power asymmetry/relationship between nurse/doctor and patient, a dynamic that is also observed in other areas of society (2). In humanist healthcare, the unequal doctor–patient relationship is common, with the doctor exercising control over the patient on the basis of rational scientific authority (3). Nursing and medicine are also permeated by such power relations (medical paternalism) (8).

At the same time, also the clearly humanist value of medicine is the non-acceptance of correctable defects and disabilities. The healthcare promotes by using ever more advanced technical means to compensate for these visible disabilities, for example. Moreover, cosmetic and gender-affirming surgery has long rendered humans malleable (9), making it possible to correct a condition that is not personally acceptable—a concept that is rooted in humanist terms.

The posthumanist approach to health does not seek to dehumanize; rather, it integrates the human and the non-human (decentralizes) (6). Posthumanist nursing signifies openness to desires and ways of being beyond the walls of the clinical environment. While the hospital isolates the patient's body, posthumanist care opens up to the hybrid nature of reality (10). For example, the use of virtual reality glasses by dementia patients enables them to inhabit the reality they create and escape into cyberspace (11). This blending—cyborg existence—reflects the fact that many humans possess bodies that are chemically, surgically, and technologically modified and live in close contact with machines is this vision of posthumanism. A new phase of human evolution is possible, characterized by the blurring of the boundaries between humans and non-humans.

## A critique of the posthumanist health conception

Posthumanist theory attempts to resocialize and decentralize the human subject. In healthcare, we decentralize ourselves through the use of medical devices, but at the same time we immediately subordinate ourselves to the control of digitalization. Even the simplest mHealth (mobile Heath) devices are already embedded in the physical self-image of many people (12, 13). One example is nomophobia (a disease), defined as the anxiety experienced by those who do not have a working smartphone (14). According to P. Standbrink, the non-actionable, biologically exogenous phenomenon of commodity-cyborgism [the use of artificial intelligence (AI)-powered smart devices] is also a form of cyborg existence (15). Posthumanism in health technology—with its decentralization of the subject—conceptualizes the body as part of a complex techno-ecosystem (4), thereby making the definition of the human more malleable.

However, posthumanist healthcare does not provide answers to many questions left unanswered by humanism and even generates new questions. Posthumanism brings up the topic of inequality. I don't see how posthuman healthcare solves the problem of inequality in health care, why does not becoming a medical human-cyborg getting a matter of material resources, for example? It may alter the dynamics in doctor–patient relationships, but the fundamental problem remains the ability of the patient to gain health information. The patient (e-patient) can participate—as an equal partner—in matters concerning his/her own illness (no decision about me, without me). However, it is increasingly difficult to discern the truth of the information one obtains: Fake news has permeated the healthcare sector (16). People also lack critical thinking and source-criticism skills to recognize health-related misinformation.

Generative artificial intelligence and large language models can increasingly help in obtaining health information (17), but only if used discerningly by the e-patient, which is the privilege of a few. This in itself constitutes inequality, as the majority of people cannot keep pace with the IT advances needed to manage such tools (18).

## The definition of a cyborg

A cyborg is defined as a creature that combines mechanical (cyber) and biological (organic) components. In art (movies, books, comics), cyborgs are mostly portrayed as super-powered creatures equipped with sensors, weapons, robotic limbs, or even AI systems to help them make decisions, with the key aspect being that they are amplified and have superpowers. Most of us first heard the term cybernetic organism (cyborg) through the Terminator 1 movie (T-800). But the Terminator is not actually a cyborg in the current (or future) medical sense. Its organic parts were just the skin and muscles, designed to blend in with humans (cover the machine). The T-800 and the more advanced version in Part 2—with human emotions—can be considered a biohybrid robot: It is an electronics system with human tissue, and in the case of the T-models, a tissue mantle. Similarly, androids or replicants are not cyborgs, as they are AI-controlled robots that do not contain human tissue, although they are fully human-like.

In reality, increasing numbers of people live with some kind of medical device or artificial organ in their body—pacemaker, prosthesis, etc. These people cannot be called cyborgs. Should we consider them cyborgs? How many incorporated electronics are required before one qualifies as a cyborg? Or does cyborg status only apply when AI plays a fundamental role in the electronics and in independent health decision-making? The implantable cardioverter defibrillator (ICD) and the pacemaker also make independent decisions, but they are not AI systems.

A medical implant is a device or tissue that is placed in or on the body. Most implants are prostheses (e.g., sense bodily functions) and mainly support organs (e.g., medical devices). A prosthesis is an artificial/engineered device that replaces a limb or missing organ. A prosthesis is an artificial replacement part of the body, replacing a part that is missing or no longer functioning properly. An artificial heart—as a highly technical medical solutions—that can be used now e.g., to go to school is just a more serious implant only? (19) Or is a person with such an artificial heart living as a human-cyborg? A cyborg can therefore also be a combination of a human and a machine: part human, part robot, signifying the medical human-cyborg.

Many people have attempted to classify medical human-cyborgs. Broadly, they are classified according to the different levels of integration of artificial accessories into the body. For example, one category includes humans with neurological devices (e.g., bionic arm) (20), while another encompasses humans with devices that supplement or replace the nervous system (e.g., cochlear implantation). Some classifications even introduce categories in which the devices add a new function to the nervous system, such as reconfigurative, adaptive, or creative, implying machine learning capabilities within the device (21). According to P. Standbrink, cyborgism in the sense of human development (machine and digital) takes seven forms, differing in intensity, extent and degree of hybridity (15). In his framework of biologically embedded medical human-cyborgism, he mentions two degrees: biologically exogenous medical human-cyborgism (e.g., exoskeleton) and biologically implanted medical human-cyborgism (e.g., totally artificial heart, TAH). He also identifies a category for non-actionable biologically exogenous commodity-cyborgisms (e.g., smartphone, interactive digital platforms). Other publications consider any technical or mechanical device that is connected to the body—to assist human hearing, movement, vision, or failing organs—as an example of medical human-cyborgism (22).

However, there is no clear definition of the human-cyborg condition. One could even argue whether one can be called a cyborg on the basis of how many artificial organs one has implanted; for example, how what combination of ICD, cochlear implant, pacemaker, and prosthesis is required to qualify for medical human-cyborg existence? Alternatively, should the degree of machine–human hybridity (more machines with essential-to-life functions and less human share) be a measure of medical human-cyborg existence?

## Conclusion

There is, as yet, no accepted definition of a human-cyborg. In my opinion, with the significant progress of medicine, such a definition seems to be necessary. The definition should declare that the cyborg concept defines exclusively physiological functional capacity, or attempt to integrate this medical definition into the philosophical, cognitive literature approach. In my view, people with implants and artificial organs are not yet medical human-cyborgs; rather, they represent great achievements of humanistic medicine. Healthcare currently produces repaired patients with technological, bionic, and AI-based interventions through machines. As long as the abilities of the treated people do not differ from those of healthy people, we can only consider this healing from the technological development of healthcare (“Repaired Man”). For example, a person who sees with a bionic eye does not see better, a person with a bionic arm is not stronger, and a person with a TAH is not more fit than a healthy person.

However, the integration of the machine (+AI) into the human body may reach such an extent (a degree of hybridity) that it will not only be a supplementary tool for the nervous system but will also be suitable for machine learning. If the technology reaches the point when the patient with the artificial organ is able to do more than a healthy person, then the “Man with Extras” is created. This development not yet means that humanist healthcare has reached the point of the patient's dependence and intertwining with medical technologies. Rather, it means that medical science has begun to actively control abilities. As long as this enhancement happens for the purpose of healing (as an advantageous side effect, just as many drugs have beneficial side effects), it may perhaps still be called humanistic medicine.

In my opinion, one can only become a medical human-cyborg if one also receives cybernetic organ superpowers compared to a healthy person. Consequently, if a sick person receives a device that not only supplements their impaired abilities but also increases biological abilities, then the medical human-cyborg. Perhaps this is when healthcare enters a posthuman state.
